# Approaching Multimorbidity From a Translational Geroscience Perspective

**DOI:** 10.1093/geroni/igab046.1339

**Published:** 2021-12-17

**Authors:** Anne Newman

## Abstract

Multimorbidity describes the accumulated burden of chronic disease. Multimorbidity erodes physiologic reserve, increasing the risk of frailty, disability and death. Most older adults have at least one chronic health condition by age 65. Once established, many age-related conditions progress and accumulate with age. Geroscience holds that there are key biologic pathways that explain the increase with age in multimorbidity, frailty and disability Translation of geroscience principles to human studies requires careful assessment of biomarkers of these pathways and multisystem outcomes. In this symposium, translational researchers in geriatric medicine and gerontology will present current work to elucidate biologic underpinnings of aging and potential intervention targets. We will address whether blood biomarkers of aging processes are prognostic using combinatorial techniques and explore the potential for proteomics to identify novel pathways for health aging. New insights into the role of inflammation will be discussed with an emphasis on its relationship to multimorbidity. Brain aging will be considered with respect to the interactions between external stressors and resilience evaluating the role of ketone bodies which have immunomodulatory effects particularly on innate immune cells. Finally, the role of multimorbidity as an intervention target and potential intermediate outcomes including biomarkers will be presented with discussion of next steps needed to realize the potential for translational geroscience clinical trials to improve health span.

